# Supplementary material for: Magnetic omniconversion: Source-independent molding of magnetostatic fields
Source: Sci Adv. 2026 Oct 9;12(41):eaeh5747. doi: 10.1126/sciadv.aeh5747 (PMC13651880; doi:10.1126/sciadv.aeh5747)
Supplement: Supplementary file 1 [file sciadv.aeh5747_sm.pdf]

Science Advances
AAAS

Supplementary Materials for

Magnetic omniconversion: Source-independent molding of magnetostatic fields

Jaume Cunill-Subiranas *et al.*

Corresponding author: Jaume Cunill-Subiranas, jaume.cunill@uab.cat; Witlef Wieczorek, witlef.wieczorek@chalmers.se; Carles Navau, carles.navau@uab.cat

Sci. Adv. **12**, eaeh5747 (2026)
DOI: 10.1126/sciadv.aeh5747

This PDF file includes:

Supplementary Text
Figs. S1 to S6
Table S1

Experimental details

The system

The experimental setup consists of a high-purity aluminum tube (4N) with a wall thickness of 2.5 mm, an inner radius of 22.5 mm, and a length of 100 mm (see Fig. S1). The end caps are made of cast iron, each with a thickness of 6.5 mm, of which 4 mm are inserted inside the tube and 2.5 mm extend longitudinally. Throughout the experiment, the aluminum tube is positioned horizontally inside the dilution-refrigerator cryostat (Bluefors LD250), supported by press-fitting it into two brass mounts and secured with screws to the mixing stage.

The printed circuit board (PCB) used for measurements contains 11 commercial 1D linear tunneling magnetoresistance (TMR) sensors, Allegro CT100, arranged to map the axial component of the field across different regions of the tube. In Fig. S2 (left), the two central-bottom ports are used to connect the supply voltage cables for all sensors, while the remaining 22 cables are used to record measurements depending on the sensor being monitored. The sensors were soldered onto the PCB using lead-free solder paste.

The board is placed horizontally inside the tube, with its dimensions (90 mm $\times$ 45 mm) such that the tube itself acts as a support, securing it in place to prevent movement. The aluminum tube has a 1 mm thick slit running the entire length, parallel to the tube axis. Through this slit, the 24 copper wires pass.

A multi-turn NbTi coil is used as the magnetic field source. It is mounted near the aluminum tube on a 3D-printed spool, leaving a 12 mm gap between the coil and the tube. This spacing prevents exceeding the critical field of aluminum, which might occur if the coil were wound directly onto the tube. Throughout the experiments, the coil is always positioned in the same location, near one end of the tube (adjacent to one of the brass mounts, as in Fig. S2).

Data processing

Raw data. To carry out the measurements of the magnetostatic field, the voltage values measured by each of the 11 sensors on the PCB were recorded as a function of the current flowing through the NbTi coil. This procedure was repeated under different conditions in order to obtain the four distinct cases presented in Fig. 3 of the main article.

In Fig. S3(a), we show the absolute values of the recorded voltages when the system is at room temperature and the cast-iron caps are not in place. Each data point represents the mean of five consecutive voltage measurements. The numbering of the sensors follows the order from left to right and from top to bottom, as shown in Fig. 3. For the case shown in Fig. S3(b), where the caps are mounted on the tube ends at room temperature, we also plot the corresponding average voltages.

This same system was measured at temperatures below 1.0 K (zero-field cooling), allowing the high-purity aluminum tube to enter the superconducting state. Although the sample itself lacked a temperature sensor, we monitored the temperature using the mixing-stage sensor of the cryostat. The results are shown in Fig. S3(c) without the cast-iron caps and in Fig. S3(d) with the caps present.

However, since the temperature difference between the measurements performed at room temperature and those at cryogenic temperatures is large and slightly affects the sensors, the voltages were also recorded at a cryogenic temperature of (1.28 ± 0.01) K, which is above the critical temperature of aluminum. These measured values are shown in Fig. S4(a) and allow a correction factor to be applied to the voltages measured at room temperature for comparison with those obtained at low temperatures.

Linear fits. By performing a linear fit of the sensor voltages as a function of the coil current (which is proportional to the applied field), the slopes of each linear regression can be obtained. These slopes are directly proportional to the field experienced by each sensor relative to the applied field. The slopes resulting from these linear regressions are normalized and shown in Table S1. All correlation coefficients from the fits range between 0.996 and 0.999990. Voltages measured at room temperature were corrected by multiplying each sensor's values by the mean of the ratios between its voltages at each coil current in Fig. S4(a) and the corresponding voltages in Fig. S3(a), allowing direct comparison with the cryogenic measurements. These mean ratios (i.e., correction factors) range from 0.97 for sensor 9 to 1.62 for sensor 1, indicating that although the effect of such large temperature changes on the sensors is noticeable, it remains moderate.

Transition to superconductivity

In this experiment, the critical temperature of high-purity (4N) aluminum was also measured indirectly, using the TMR sensors to detect the differences in the magnetic field when the tube is superconducting or not. To this end, the configuration with the PCB and sensors placed inside the aluminum tube, but without the cast-iron caps, was used. The voltage recorded by sensor number 11 was measured while driving currents through the superconducting coil. These measurements were repeated over a temperature range from 0.04 K to 1.24 K, in increments of 0.04 K. The results are shown in Fig. S4(b).

Up to 1.00 K, no significant changes are observed in the slopes of the voltage-versus-current curves for sensor 11. However, between 1.00 K and 1.12 K, the curves show a clear increase in the magnetic field at the position of sensor 11 (located near the coil). Above 1.12 K, the linear fits no longer exhibit noticeable changes, while maintaining a slope clearly higher than that observed below 1.00 K. Since sensor 11 is located near the coil, when the aluminum becomes superconducting, the magnetic field decreases in that region, as it redistributes more uniformly along the tube. However, once the critical temperature of the material is exceeded and it ceases to behave diamagnetically, the field at positions close to the NbTi coil increases. Because the temperature was monitored using the mixing-stage sensor rather than a sensor on the sample—and thus does not directly measure the material's temperature—our data constrain the superconducting transition of this 4N bulk aluminum to the range 1.00-1.12 K.

This phenomenon is relevant for the experiment, since the PCB connections are not superconducting. Consequently, when performing a current ramp, the sensors heat up and increase the temperature of the whole system. However, the ramp can be carried out quickly enough to avoid breaking the superconductivity of the aluminum tube within a single measurement. If this is not taken into account and the measurement is performed slowly while maintaining a continuous bias current through the PCB, field profiles such as that shown for sensor 1 in Fig. S4(c) can be obtained: the tube is initially superconducting, but above 50 mA, due to heating accumulated during previous measurements, a sudden change in slope (i.e., magnetic field) is observed as the critical temperature is exceeded.

About the TMR sensors

The sensors soldered onto the PCB and used to measure the magnetostatic field inside the tube are commercial devices: Allegro, model CT100 (6-lead SOT-23 package).

Each of these sensors measures a single component of the magnetic field. In this experiment, to simplify the demonstration, all sensors were oriented along the axial direction of the tube, since this is the direction of the uniform field to be generated, and the component of the magnetic field in this direction is expected to be the largest. The layout (pattern) of the sensors on the PCB was chosen to provide representative measurements of the field uniformity inside the tube and along its axial direction, using a total of 11 sensors. To obtain a more complete mapping and increase the spatial resolution, a larger number of sensors would be required.

Throughout the entire experiment, each sensor was supplied with a voltage of 3 V, which lies within the operating range recommended by the manufacturer (between 1.0 V and 5.5 V). The magnitude of the applied magnetic fields is also well within the linear operating range of the sensors. According to the datasheet, they exhibit linearity over a range of ±20 mT, with an associated error of ±0.5% within this range, which fully includes the fields measured in this work (on the order of 0.1 mT). In addition, although the manufacturer specifies an operating temperature range from −40°C to +150°C, we verified that the sensors maintain good linearity even at cryogenic temperatures. Two initial tests were performed: first, by immersing a sensor in liquid nitrogen, and second, by placing it in a 4 K cryostat. In both cases, proper operation was confirmed. Finally, as demonstrated by the results presented in this work, these sensors also operate down to millikelvin temperatures, providing excellent linearity for the measured field values. This observation is particularly relevant for applications of TMR-based sensors in cryogenic or superconducting environments, where reliable magnetic field measurements are required at very low temperatures. Our results demonstrate that this commercial sensor operates reliably under such conditions.

Discussion on materials and manufacturing

Although pure aluminum (a low-temperature superconductor, LTS) was used in this work, similar results should be achievable with high-temperature superconductors (HTS). However, the practical

realization of the system could become more challenging due to the brittleness and fabrication constraints typically associated with HTS materials. Alternatively, superconducting tapes could be employed as surface layers instead of bulk elements, as long as superconductivity is ensured.

From a manufacturing perspective, alternative materials and fabrication techniques could be explored for future implementations. For instance, lost-wax (cire perdue) casting could offer certain advantages over the use of machining for producing more complex geometries, as well as for achieving higher critical temperatures and critical fields, while additive manufacturing approaches—such as laser sintering of tantalum or other superconducting metals—could enable high-precision fabrication of intricate components compatible with cryogenic operation.

Regarding the ferromagnetic components—which could also be implemented in the form of foils or thin sheets—some hysteresis effects may arise in the cast-iron end caps, although no significant influence was observed under the quasi-static, low-field conditions of the present experiment. Cast iron was, in fact, chosen because it combines very low coercivity with high permeability and linear behavior within the applied field range (well below magnetic saturation). Other soft ferromagnetic materials, such as mu-metal or permalloy, could also serve as suitable candidates to achieve equivalent properties for the IMP surfaces.

Robustness in realizing the desired field

To study how different parameters affect the accuracy of the realized field, we performed several parameter sweeps and quantified the resulting changes in the magnetic-field-magnitude distribution within the target volume. The parameters studied were the permeability of the materials defining the boundary conditions, the position of the magnetic source, the geometry and orientation of the magnetic source, the aspect ratio of the volume where the desired field is generated, the type of target field inside the desired region, and the slit width.

To begin with, a parameter sweep of the magnetic permeabilities was carried out for the same geometry as that used in the experiment, with the slit omitted. The calculations were performed using ferromagnetic relative permeabilities of $\mu_{\mathrm{FM}} = 10^0, 10^1, ..., 10^6$ together with superconducting relative permeabilities of $\mu_{\mathrm{SC}} = 10^0, 10^{-1}, ..., 10^{-6}$, respectively. Each simulation, therefore,

corresponds to a different value of n, defined by

$$\log_{10}(\mu_{\mathrm{FM}}) = \log_{10}(1/\mu_{\mathrm{SC}}) = n\ , \qquad n = 0, 1, ..., 6\ . \tag{S1}$$

As the two permeabilities are varied simultaneously, this sweep characterizes convergence to ideal IMP/ZMP rather than the independent sensitivity to each material permeability.

To quantify the deviation from the reference solution for a given pair of permeabilities, we evaluated the normalized root-mean-square deviation (NRMSD) of the magnetic-field-magnitude distribution with respect to the reference case corresponding to $n = 6$. For a given value of n, this deviation is defined as

$$\mathrm{NRMSD}_n = \frac{1}{\mathrm{RMSD}_0}\sqrt{\frac{1}{N}\sum_{i=1}^{N}\left(|\mathbf{B}_{i,n}| - |\mathbf{B}_{i,6}|\right)^2}\ , \tag{S2}$$

where i denotes a point inside the target volume, $|\mathbf{B}_{i,6}|$ is the magnetic-field magnitude at that point for the reference case ($n = 6$), $|\mathbf{B}_{i,n}|$ is the field magnitude for the permeability corresponding to n, and N is the total number of evaluated points inside the target region (typically around 5000, depending on the simulation). The factor RMSD_0 is the unnormalized root-mean-square deviation between the $n = 0$ and $n = 6$ solutions for the same geometry. Consequently, $\mathrm{NRMSD}_0 = 1$ for every configuration. This metric therefore measures the average change in the spatial distribution of the magnetic-field magnitude throughout the target volume with respect to the ideal case when the permeabilities of the boundary-condition materials are modified.

The same permeability sweep was also performed for several additional configurations to assess the robustness of the omniconverter to finite material permeabilities under changes in the source position, source geometry and orientation, cylinder aspect ratio, and target field. Besides the dimensions of the experimental setup, but without the slit (blue curve in Fig. S5), we considered a geometry in which the external coil was moved away—1/3 of its radius—with respect to the cylindrical target region, thereby modifying the source position (orange curve in Fig. S5); a configuration employing a smaller-radius coil—1/5 of its original radius—that was both displaced and tilted—$\pi/6$ radians—with respect to the cylinder axis, thereby modifying the source geometry and orientation (green curve in Fig. S5); a geometry in which the length of the target cylinder was reduced by a factor of two, thereby changing its aspect ratio (red curve in Fig. S5); and finally a configuration targeting a dipolar field, as in Fig. 2(c) of the main manuscript (purple curve in Fig. S5).

In every case, the NRMSD was computed with respect to the corresponding $n = 6$ simulation of the same geometry.

Figure S5 shows that the permeability of the boundary-condition materials has a significant influence on the deviation from the ideal field, primarily for relatively small n's, as expected. In all the studied configurations, the NRMSD decreases rapidly as n increases, indicating that the obtained field quickly converges toward the ideal solution as the ferromagnetic and superconducting materials approach their ideal magnetic properties. For $n \geq 3$, the NRMSD remains below 20% for all the studied configurations and continues to decrease for larger values of n.

Although the different configurations exhibit different rates of convergence, all of them show the same general trend, demonstrating that the realization of the desired field is robust for finite material permeabilities provided that sufficiently large n's are achieved.

Next, a parameter sweep was performed to study the effect of the slit in the experimental geometry. In this case, the permeabilities were kept fixed at $n = 6$ for all the simulations, and the only parameter that was varied was the slit width, s (in mm), which ranged from 0 mm to 6 mm. The reference case was taken to be the cylinder without a slit ($s = 0$), and the deviation was evaluated using an expression analogous to Eq. (S2),

$$\mathrm{NRMSD}_s = \frac{1}{\mathrm{RMSD}_1}\sqrt{\frac{1}{N}\sum_{i=1}^{N}\left(|\mathbf{B}_{i,s}| - |\mathbf{B}_{i,0}|\right)^2}\,. \tag{S3}$$

The resulting RMSD values were normalized by the RMSD corresponding to a slit width of 1 mm—as in the experiment. The results of this parameter sweep are shown in Fig. S6.

Figure S6 shows that increasing the slit width progressively increases the deviation from the slit-free reference field. Since the slit-free configuration produces the most homogeneous field, this trend is consistent with a progressive reduction in field homogeneity inside the cylinder.

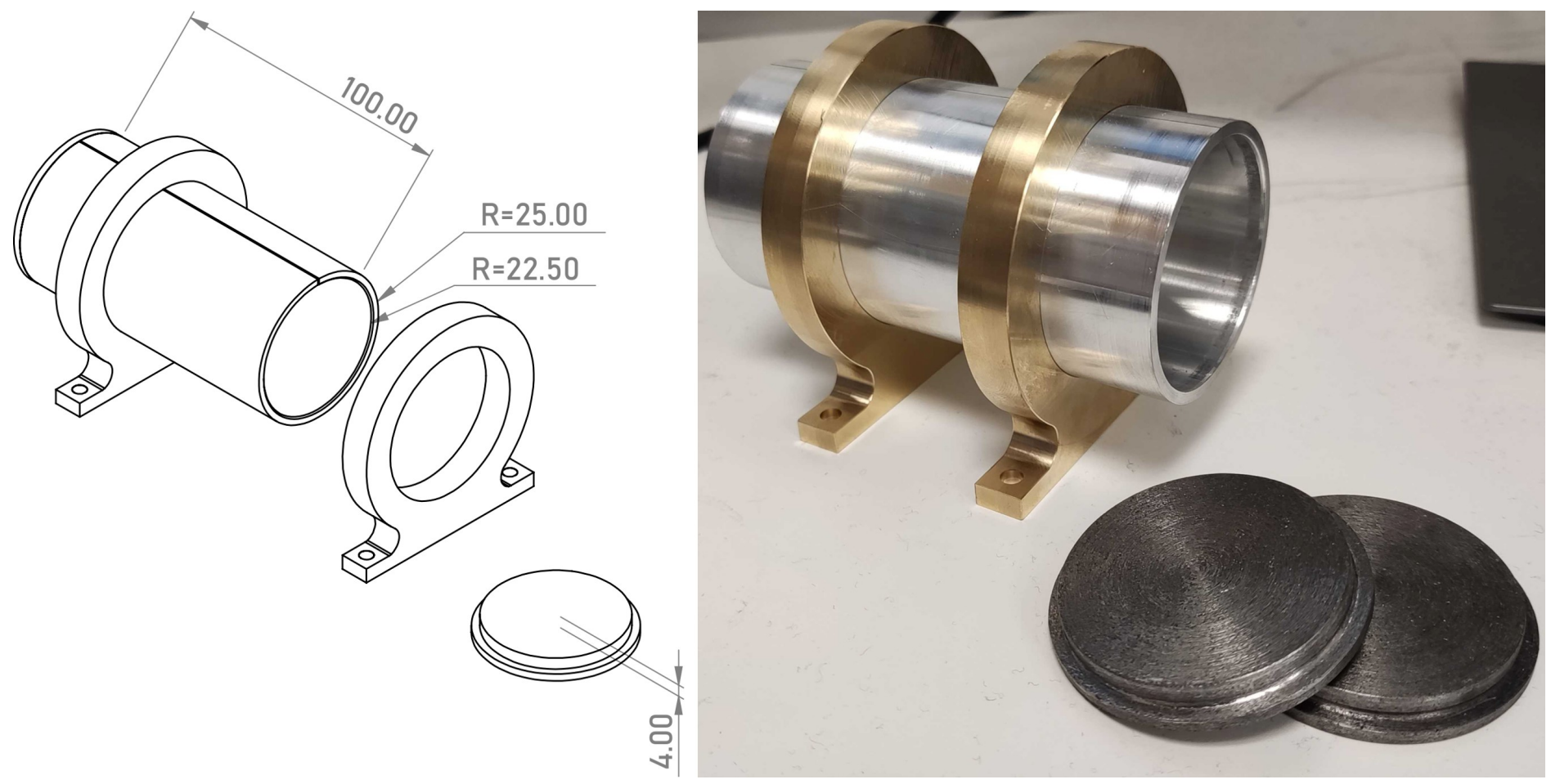

Figure S1: **Geometry of the aluminum tube assembly.** Dimensions (in mm, left) and photograph (right) of the aluminum tube with two cast-iron end caps and two brass supports.

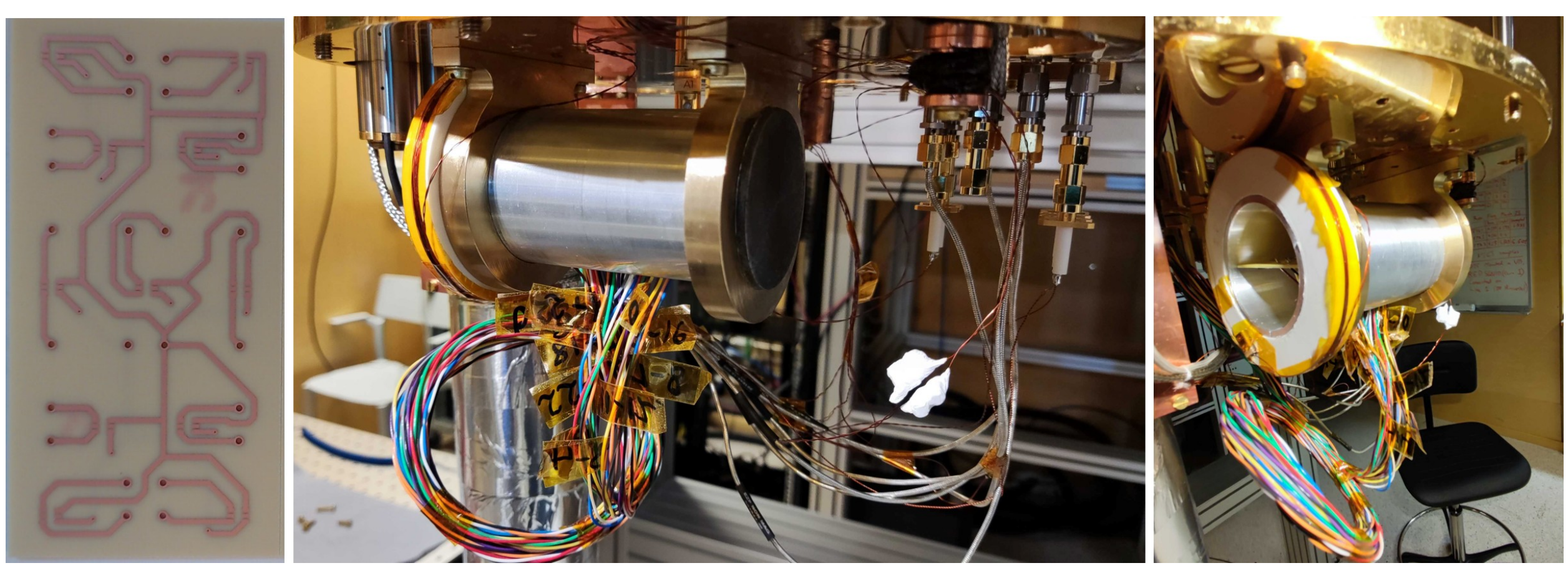

Figure S2: **Photographs of the PCB and final assembly within the cryostat.** Photographs of the PCB before soldering the commercial Allegro CT100 sensors (left), and the complete experimental assembly integrated into the cryostat, shown with caps (center) and without caps (right).

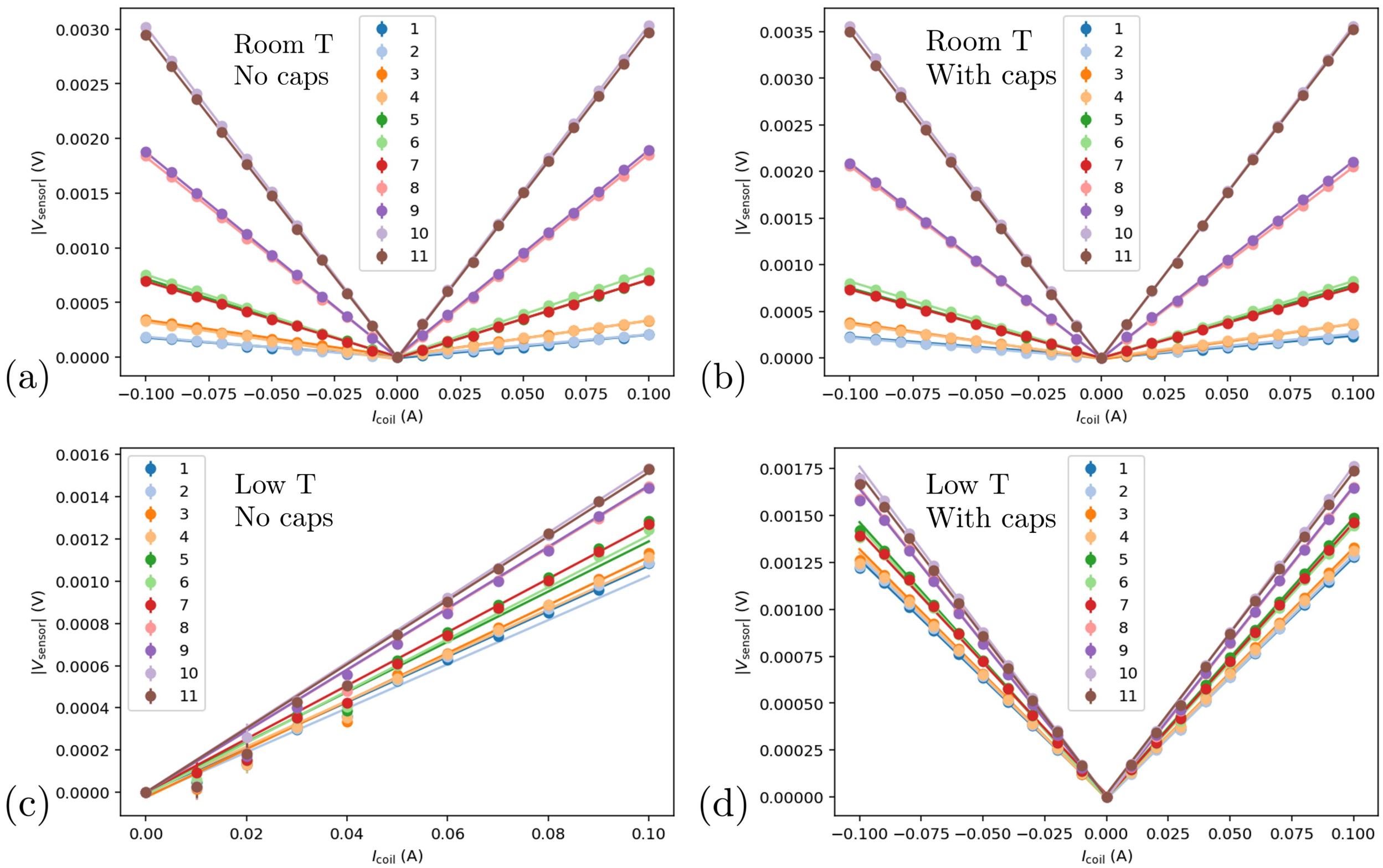

Figure S3: **Experimental measurements.** Absolute values of voltage measured at each of the 11 TMR sensors as a function of the current flowing through the NbTi coil. Each data point represents the mean of five consecutive voltage measurements. Solid lines are linear fits and, for most points, error bars are smaller than the symbol size. (a) System at room temperature and the cast-iron caps are not in place. (b) System at room temperature and the cast-iron caps are mounted on the tube ends. (c) System below 1.0 K and the cast-iron caps are not in place. (d) System below 1.0 K and the cast-iron caps are mounted on the tube ends.

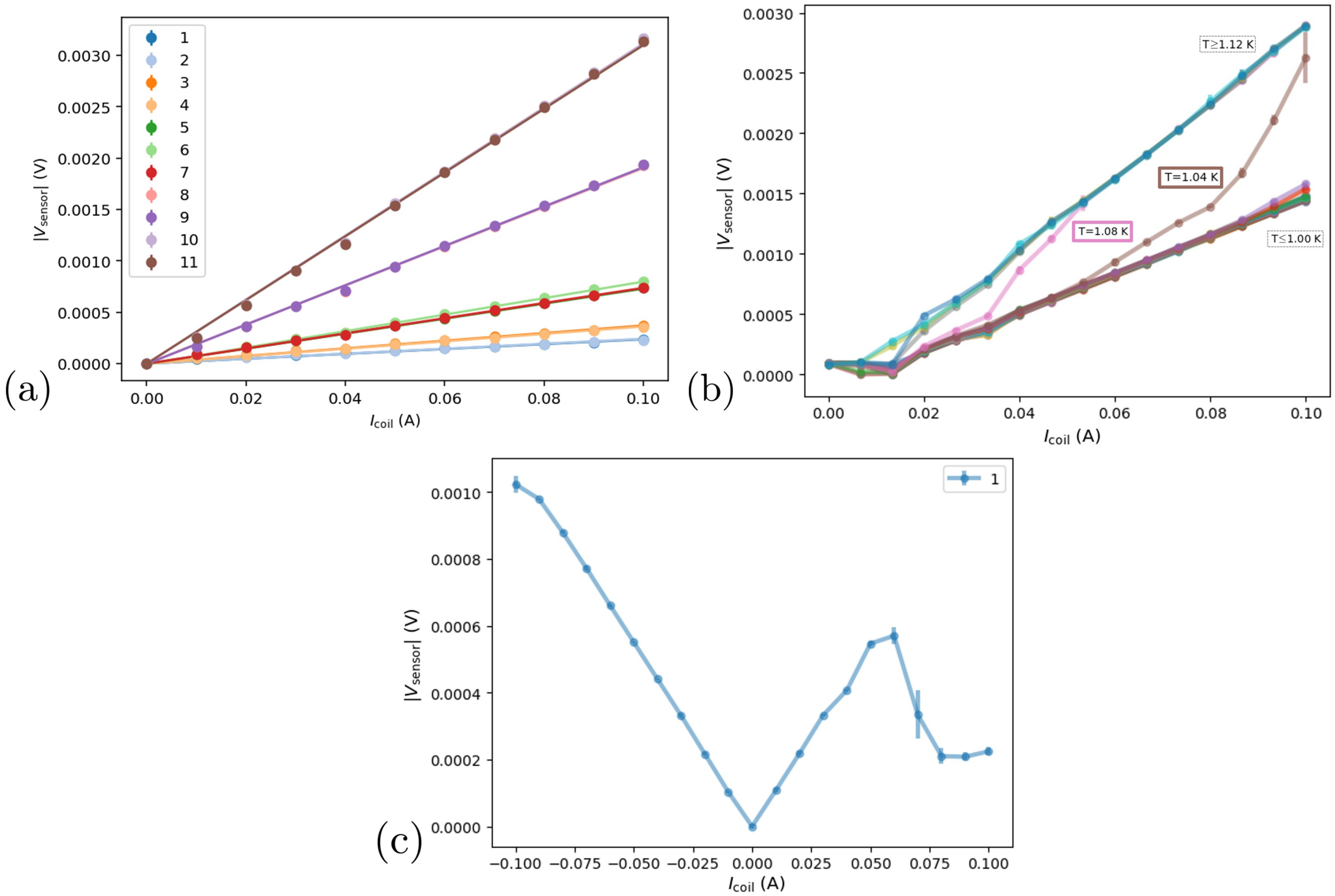

Figure S4: **Temperature effects while measuring.** Absolute values of voltage measured for some of the TMR sensors as a function of the current flowing through the NbTi coil. Measurements were performed without the cast-iron caps in the tube. Each point represents the mean of five consecutive measurements [with a 0.25 s and 0.50 s interval between them in panels (a,b) and (c), respectively]. For most points, error bars are smaller than the symbol size. (a) Voltages measured by the 11 TMR sensors at a cryogenic temperature of (1.28±0.02) K, above the critical temperature of aluminum. Solid lines are linear fits. (b) Voltage measured by sensor number 11 over a temperature range from 0.04 K to 1.24 K in 0.04 K increments. Solid lines are guides for the eye. Uncertainty on all temperatures: ±0.02 K. The overlapping upper lines correspond to temperatures between 1.12 K and 1.24 K, while the overlapping lower lines correspond to temperatures between 0.04 K and 1.00 K. In these ranges, no change in magnetic field is observed, showing that the aluminum is superconducting below 1.00 K and in the normal state above 1.12 K. In contrast, measurements at 1.04 K and 1.08 K show a change in voltage slope, evidencing the superconducting transition. (c) Voltage measured by sensor number 1 *starting* below the critical temperature of aluminum (<1 K). Solid lines are guides for the eye. The measurement was performed without temperature control during acquisition.

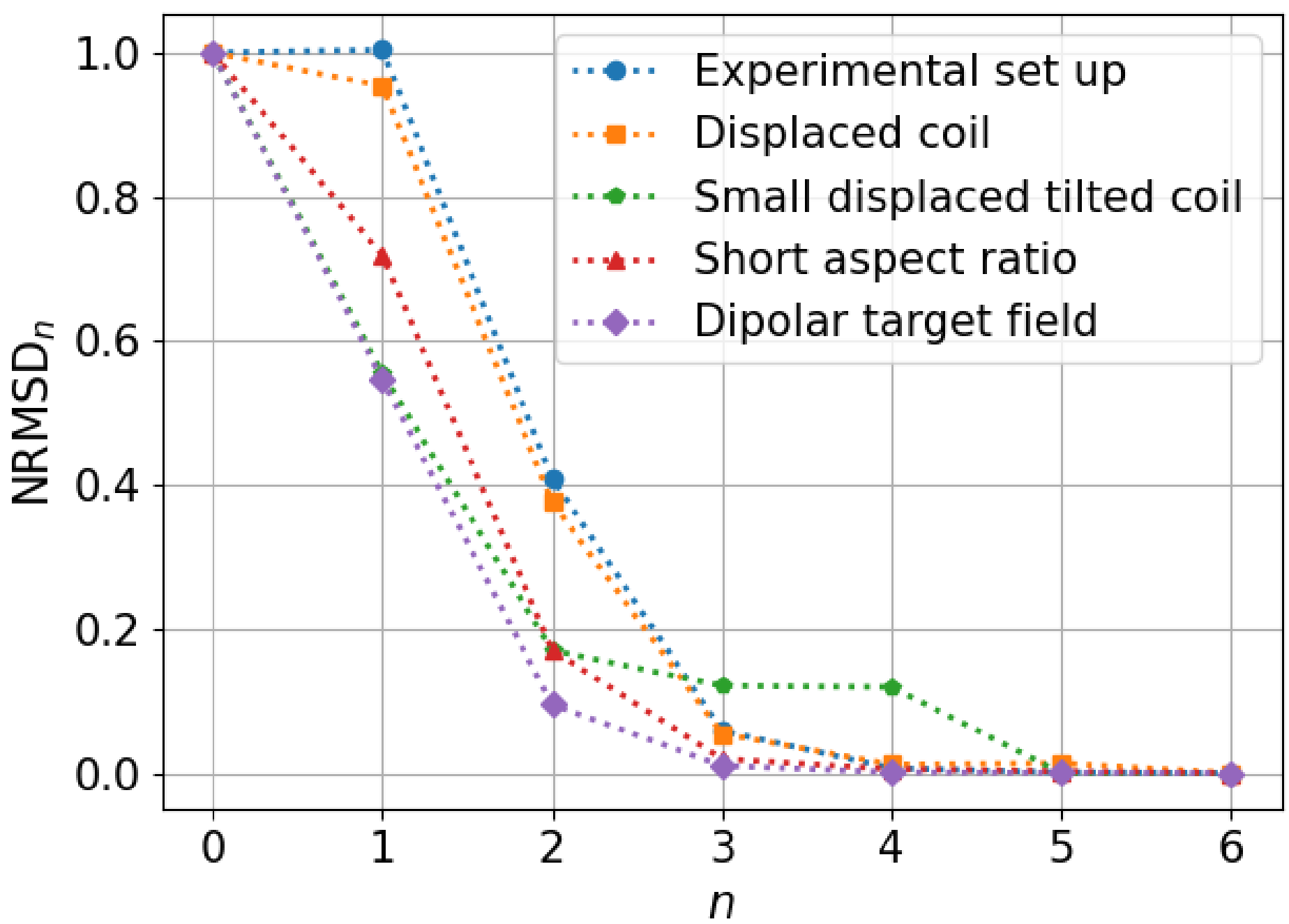

Figure S5: Normalized root-mean-square deviation, NRMSD$_n$, of the magnetic-field-magnitude distribution inside the target region with respect to the reference case ($n = 6$) as a function of the permeability index n. Each curve corresponds to a different geometry or target-field configuration. For each configuration, the unnormalized RMSD between the $n = 0$ and $n = 6$ solutions was used as the normalization factor, ensuring that NRMSD$_0$ = 1.

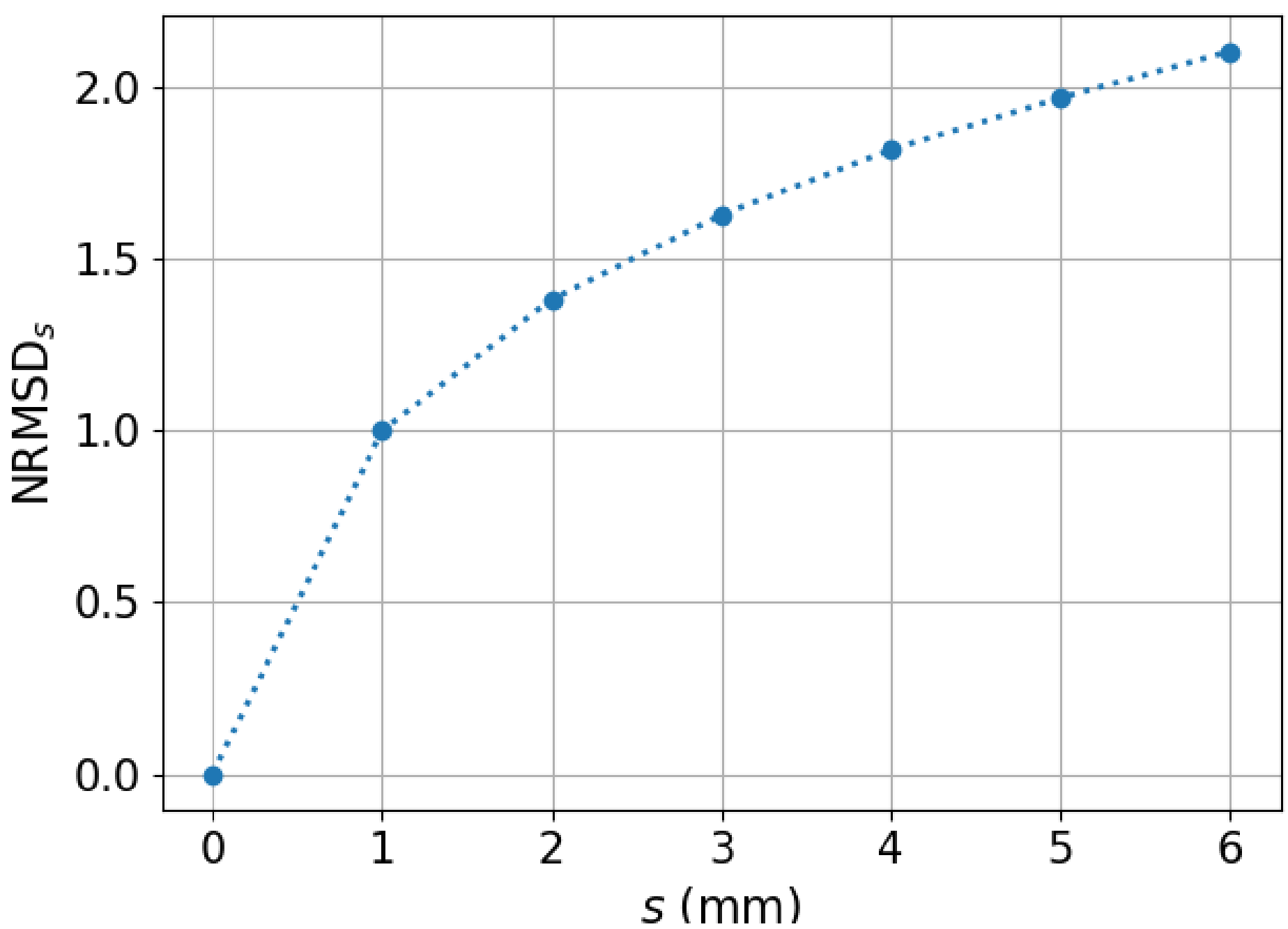

Figure S6: Normalized root-mean-square deviation, $NRMSD_s$, of the magnetic-field-magnitude distribution inside the target region with respect to the reference solution without a slit as a function of the slit width, s (in mm). The deviation was calculated using Eq. (S3), taking the magnetic field obtained for the cylinder without a slit ($s = 0$) as the reference solution. The unnormalized RMSD obtained for a slit width of 1 mm was used as the normalization factor, such that $NRMSD_1 = 1$.

Table S1: **Normalized magnetic field results.** Values of $B_z/B_{z,\max}^{\exp}$, derived from linear-fit slopes obtained from the data points in Fig. S3(a-d), respectively. A correction factor derived from data in Fig. S4(a) was applied to the (*) cases for proper comparison at cryogenic temperatures. Uncertainty on all values: ±0.010.

Sensor #	Coil*	Coil+Caps*	Coil+Tube	Coil+Tube+Caps
1	0.086	0.105	0.316	0.357
2	0.071	0.084	0.324	0.360
3	0.110	0.118	0.336	0.371
4	0.106	0.119	0.331	0.367
5	0.219	0.236	0.380	0.415
6	0.228	0.243	0.366	0.404
7	0.217	0.232	0.369	0.408
8	0.523	0.584	0.430	0.463
9	0.519	0.578	0.428	0.461
10	0.849	1.000	0.451	0.494
11	0.840	0.996	0.456	0.486
